# Supplementary material for: Simultaneous Inhibition of Ceramide Hydrolysis and Glycosylation Synergizes to Corrupt Mitochondrial Respiration and Signal Caspase Driven Cell Death in Drug-Resistant Acute Myeloid Leukemia
Source: Cancers (Basel). 2023 Mar 21;15(6):1883. doi: 10.3390/cancers15061883 (PMC10046858; doi:10.3390/cancers15061883)

Figure S1. Full western blots of AML cell lines for Figure 2E. Red line were cut out and used for the figure.

P-gp Full western blots

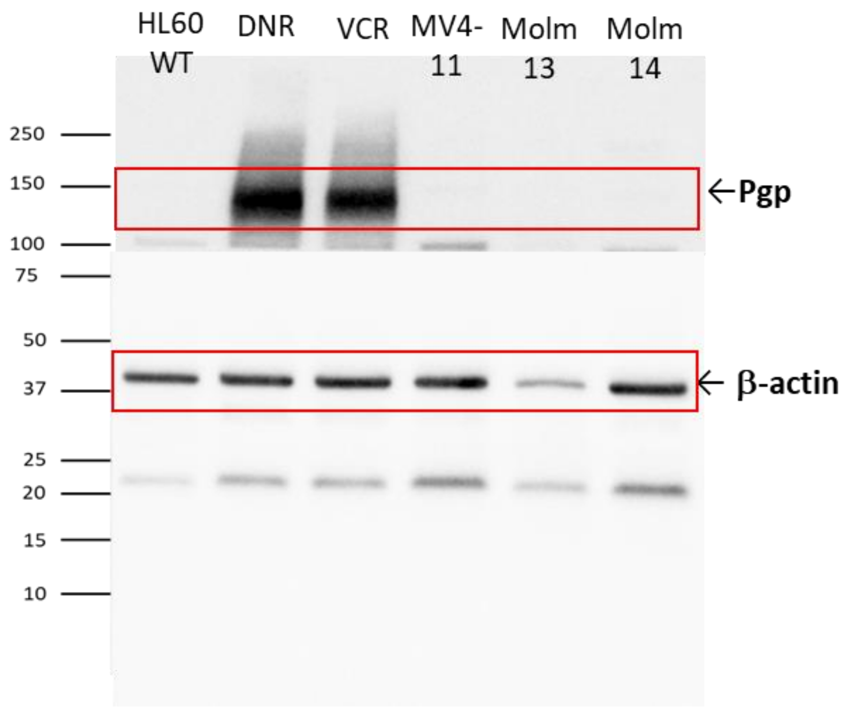

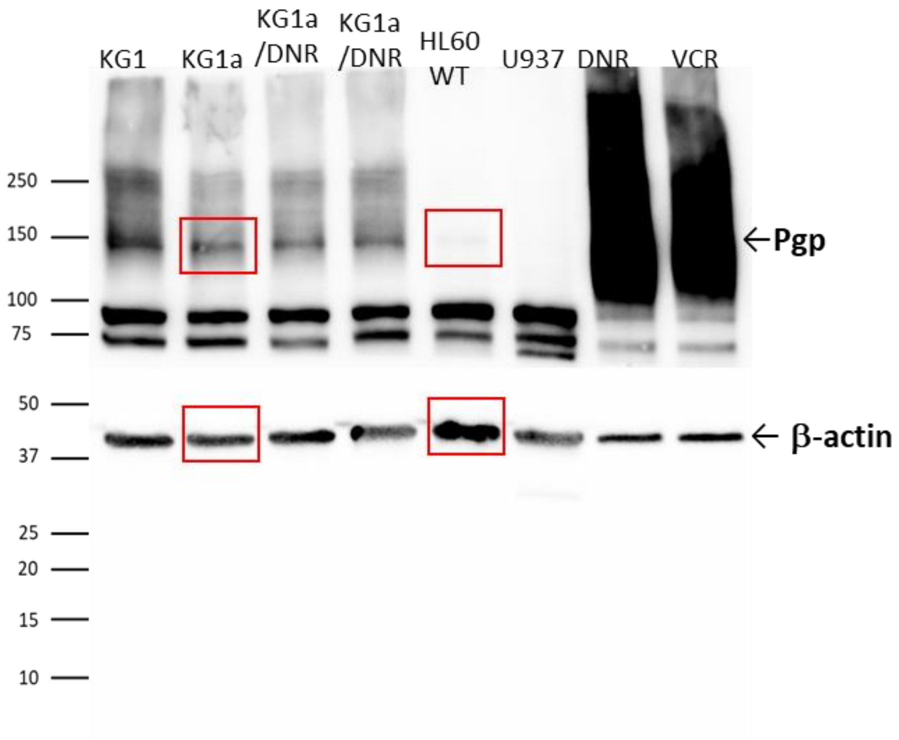

Figure S2. Full western blots of HL-60/DNR cell line treated in control and mix (SACLAC + D-*threo*-PDMP, 5 + 10 μM, except pGSK-3β was 10 + 20) for Figure 5B. Red line were cut out and used for the figure.

pAkt Full western blots

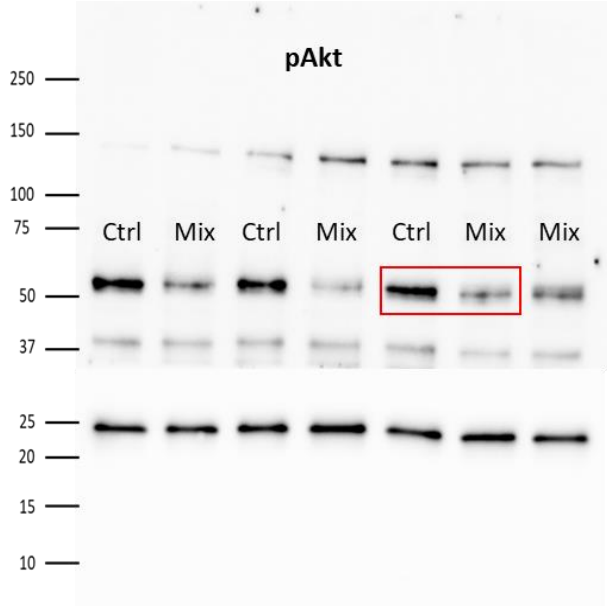

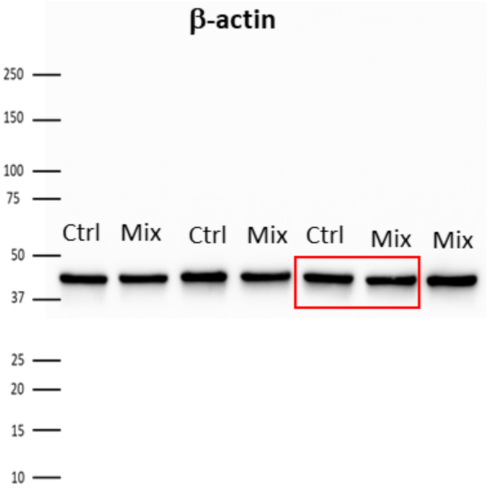

pGSK3b Full western blots

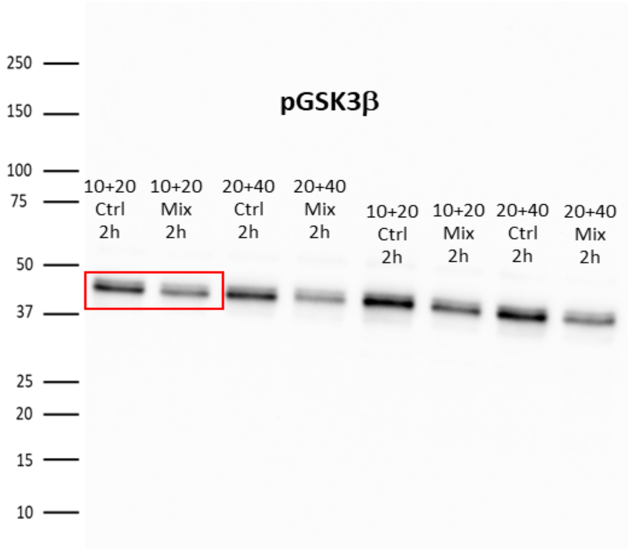

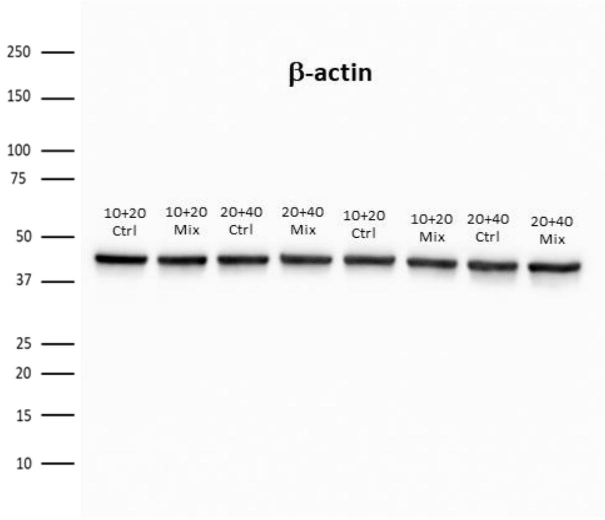

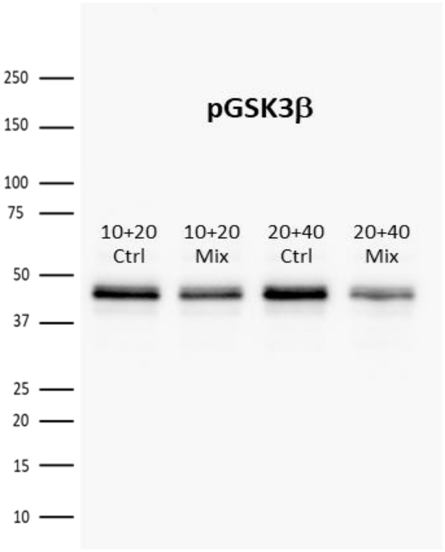

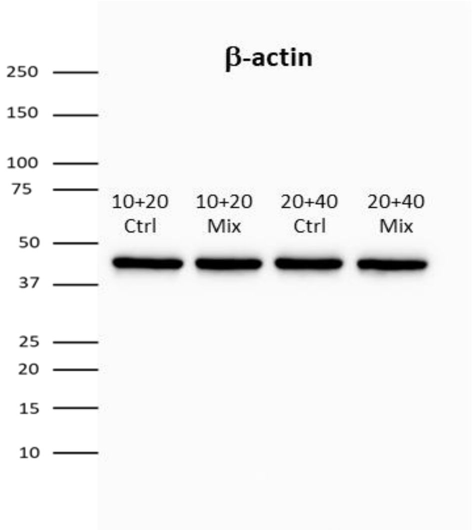

MCl-1s Full western blots

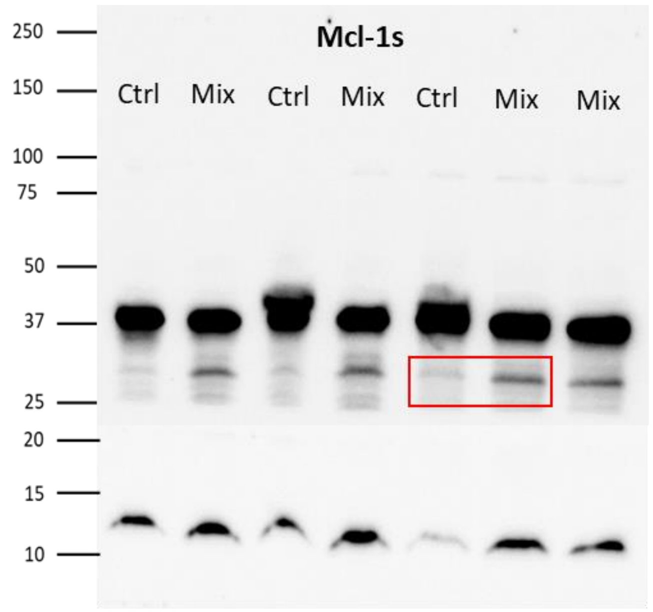

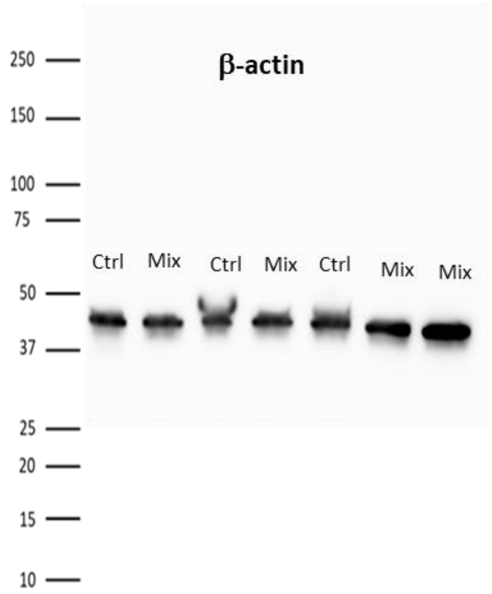

clv Cas-2 Full western blots

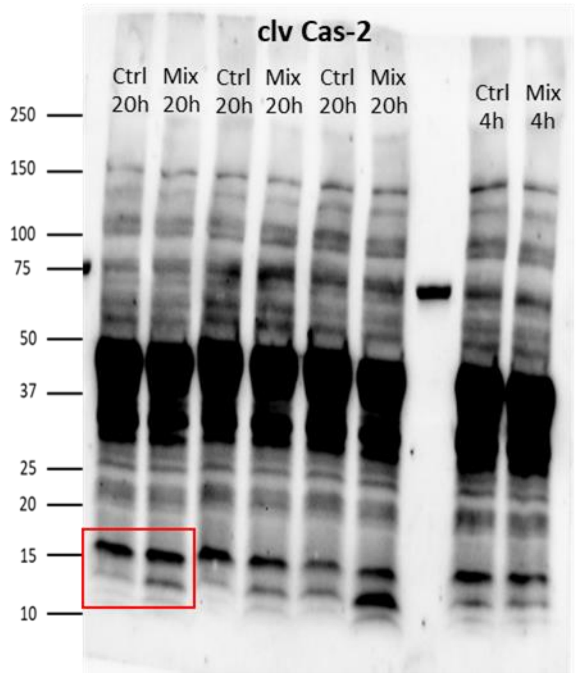

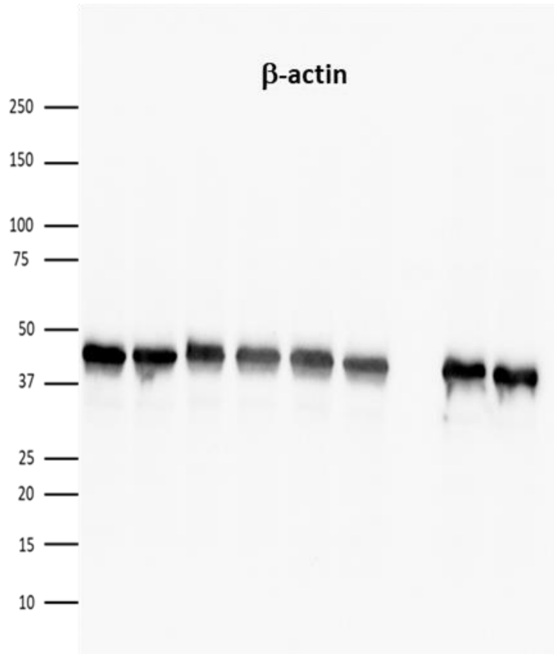

Surviin Full western blots

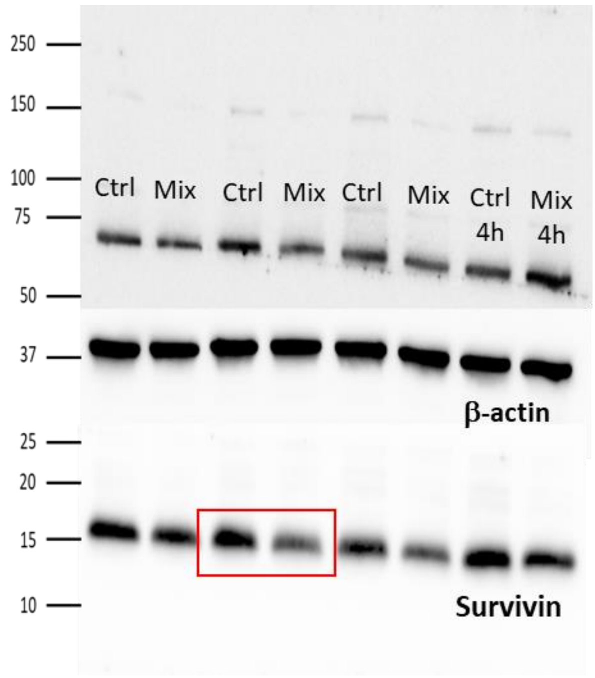

clv Cas-9 Full western blots

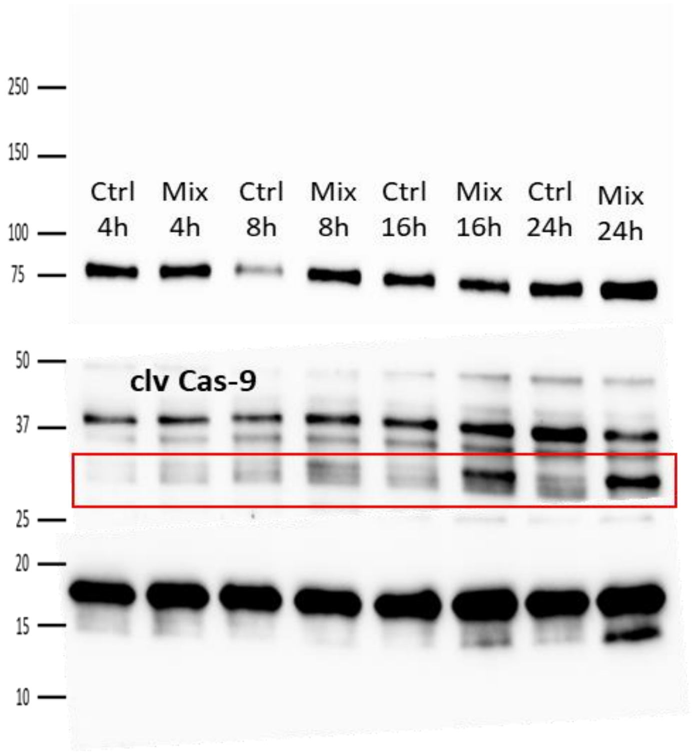

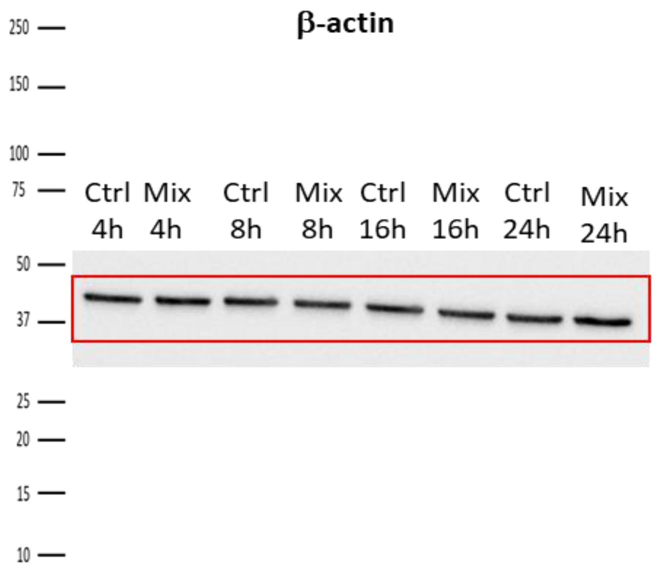

clv Cas-3 Full western blots

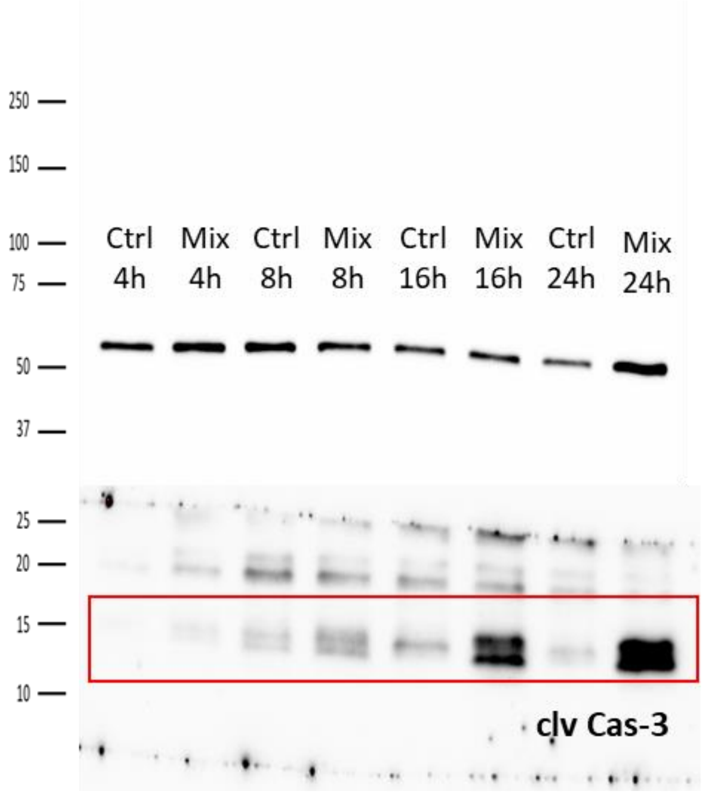

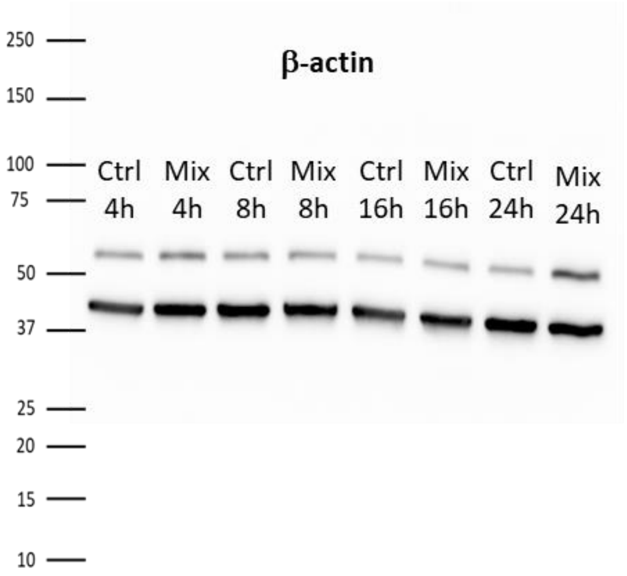

Supplement: Supplementary file 1 [file cancers-15-01883-s001.zip › cancers-2252288-supplementary.pdf]
